# SB Brasil 2023: methodological insights and key oral health findings

**DOI:** 10.1590/1807-3107bor-2025.vol39.051

**Published:** 2025-05-19

**Authors:** Andrea Maria Duarte VARGAS, Doralice Severo da Cruz TEIXEIRA, Raquel Conceição FERREIRA

**Affiliations:** (a) Universidade Federal de Minas Gerais – UFMG, School of Dentistry, Departament of Social and Preventive Dentistry, Belo Horizonte, MG, Brazil.; (b) Ministério da Saúde, General Oral Health Coordination, Braília, DF, Brazil.

Oral health surveillance initiatives serve as essential tools for evaluating programs, identifying problems, and refining strategies for both health promotion and patient care. In this context, the National Oral Health Policy, which was established as a State Policy with the sanctioning of Law 14572 of May 8, 2023, provides a strategic framework for guiding oral healthcare approaches. One of its key principles is using epidemiology to support action planning within healthcare services and to strengthen surveillance efforts.

In Brazil, four major epidemiological surveys on oral health have been conducted (1986, 1996, 2003, and 2010), contributing to the development of a comprehensive database on the population’s oral health profile. The Ministry of Health’s initiative to establish a historical series of data for oral health surveillance (SB Brasil) has proven to be highly effective. Beyond identifying trends in various oral health conditions, this strategy has played a crucial role in guiding the planning and evaluation of proposed interventions.

This issue of Brazilian Oral Research presents the first articles based on findings from SB Brasil 2023.

The methodological aspects of SB Brasil 2023 were analyzed in an article based on documentary research, comparing the methodologies used in previous editions (SB Brasil 2003 and SB Brasil 2010).^1^ The study aimed to examine the methodological changes that occurred over time, emphasizing advancements while also addressing the challenges of maintaining the consistency and reliability of this oral health surveillance strategy in Brazil.

The second methodological article presents the sampling plan, with a focus on evaluating the precision of estimates for the dmft and DMFT indices across the sampling scopes of SB Brasil 2023.^2^ The third article examined the development and application of technologies associated with the in-lux method, used in the calibration of examiners for SB Brasil 2023. It also presents the agreement coefficients achieved by the examiners for each condition assessed during training.^3^ These studies aimed to contribute to discussions on advancements and challenges in refining the methodological approach for primary data collection, offering valuable insights for future editions of SB Brasil.

The selected original studies focused on specific age groups, providing a detailed analysis of key oral health conditions.

For 5-year-old children, the study analyzed dental caries, a highly prevalent condition in this age group, and no significant change compared to the SB Brasil 2010 results was observed.^4^ Among 12-year-olds and adolescents aged 15 to 19, dental caries was also the primary condition analyzed, with a comparative assessment of the DMFT index across previous surveys.^5,6^ Additionally, the study explored regional disparities, highlighting inequalities in disease burden. Notably, for the first time, the clinical consequences of untreated dental caries were assessed on a national scale in the 12-year-old group.

Among adults, the research was focused on the access to and utilization of dental services, use of prosthesis, and both self-perceived and clinically assessed treatment and prosthetic needs.^7^ In the elderly population, the findings revealed a decline in tooth loss compared to previous surveys (SB Brasil 2003 and 2010).^8^ Given this trend, the study aimed to contextualize these improvements while considering socioeconomic factors such as education, providing insights into how changes occurred across different demographic groups and contributing to the discussion on oral health inequalities.

The studies underscore significant progress in the oral health of the Brazilian population, reflecting important achievements. However, they also highlight persistent challenges that require continued efforts, particularly in addressing the inequalities revealed by the data.

We believe that the findings presented here will be highly relevant to oral health policymakers and practitioners, as they provide a data-driven foundation for planning targeted actions aligned with epidemiological reality. Additionally, these findings offer an opportunity to further explore methodological aspects, fostering the development of new research initiatives and technological innovations. Furthermore, these findings and reflections reinforce the importance of SB Brasil as a strategic tool for oral health surveillance, strengthening the connection between scientific research and public policy management to drive more effective interventions and improve oral health outcomes for the Brazilian population.
